# What do we have to know from migrants' past exposures to understand their health status? a life course approach

**DOI:** 10.1186/1742-7622-8-6

**Published:** 2011-08-15

**Authors:** Jacob Spallek, Hajo Zeeb, Oliver Razum

**Affiliations:** 1 Bielefeld University, Faculty of Health Sciences, Department of Epidemiology & International Public Health, Universitätsstraße 25, 33501 Bielefeld, Germany; 2 Bremen Institute for Prevention Research and Social Medicine (BIPS), Department of Prevention and Evaluation, Unit of Social Epidemiology, Achterstraße 30, 28359 Bremen, Germany

**Keywords:** Migrants, migrant health, life course epidemiology

## Abstract

Empirical findings show that morbidity and mortality risks of migrants can differ considerably from those of populations in the host countries. However, while several explanatory models have been developed, most migrant studies still do not consider explicitly the situation of migrants before migration. Here, we discuss an extended approach to understand migrant health comprising a life course epidemiology perspective.

The incorporation of a life course perspective into a conceptual framework of migrant health enables the consideration of risk factors and disease outcomes over the different life phases of migrants, which is necessary to understand the health situation of migrants and their offspring. Comparison populations need to be carefully selected depending on the study questions under consideration within the life course framework.

Migrant health research will benefit from an approach using a life course perspective. A critique of the theoretical foundations of migrant health research is essential for further developing both the theoretical framework of migrant health and related empirical studies.

## Background

Both the absolute numbers of migrants as well as their proportion of the total population are increasing in western European countries and the USA. In 2005, western and central European countries hosted more than 44 million foreign-born persons [1]. The health of migrants has been extensively studied. However, studies about health differences between migrants and majority populations face a fundamental problem: a broadly accepted comprehensive and conclusive model on migrants and their health is lacking [2]. Existing concepts of migrant health, such as the healthy migrant model [3,4], the health transition model [5] or the model developed by Schenk [6], include several important factors, but do not offer a life course perspective that takes into account the influence of health-related factors acting in the different life periods of migrants [7]. In other words, they lack an explicit time axis. The question arising from this lack of a time axis is: Which factors and exposures in the life course of migrants do we have to consider in migrant studies in order to understand adequately the current health situation of migrants? To answer this question, we use the approach of life course epidemiology. Our focus is on developing a framework for epidemiological migrant studies. Other forms of migrant studies, such as qualitative studies, are not explicitly considered.

### Life course epidemiology

Life course epidemiology can be defined as the study of physical or social exposures during gestation, childhood, adolescence, young adulthood and also adult life, with the aim ofexamining their long-term effects on health or disease risk in later life [8-10]. Life course epidemiology can help to construct models of disease aetiology with an emphasis on timing (critical/sensitive periods), duration (accumulation), and temporal sequence (triggers/interactions) of exposures [10]. One concept of life course epidemiology states that adult chronic disease can be the result of biological programming during critical periods in childhood and *in utero*. Other concepts of life course epidemiology focus on analyzing the effects of accumulated exposures over a lifetime on health risks, including the temporal sequence of exposures during the life course. These different concepts are not mutually exclusive but can operate together [8].

The health of migrants is determined by additional exposures during the life course (before, during and after migration), which are not experienced by the majority population. A migrant background is frequently associated with different exposures during specific (critical) periods, for example prenatally or in childhood, as well as with different accumulation patterns and timing of exposures. A life course perspective will help to better understand the health situation of migrants and the health differentials they experience.

### Migrant health

Strictly speaking, "migrants" are persons who migrated across national borders. Frequently, their offspring are included in a broader definition of "migrants", although these persons may not have migrated themselves. The term "migrant background", comprising both groups, is increasingly used. In this article we focus on both groups: (i) migrants who migrated themselves, and (ii) on the increasing number and proportion of people with migrant background living in Europe that are the offspring of migrants or members of ethnic minorities living in the host countries for several generations.

Migrant populations are heterogeneous in terms of cultural identity, ways of living, social situation, health behaviour and health risks. In this article we try to draw some general conclusions, while appreciating that individual migrant groups differ considerably from each other. Also, we are aware that differences between migrants and the autochthonous populations of host countries have numerous reasons, ranging from genetic background to lifestyle and nutrition. Thus not all health differentials experienced by migrants can be attributed to a single factor (such as low income), and not all differences are the expression of social deprivation or exclusion [3,6]. The main emphasis of this article is on migrants who migrated from lower-income to higher-income countries. The situation of migrant groups migrating between high-income countries, for example from Western Europe to the USA or Australia, will be different in some aspects.

Migrant populations experience different environmental and social exposures compared with the autochthonous populations of the host countries which can be summarized under the term "nurture". Additionally, differences in the genetic background ("nature") might exist, due to geographic and ethnic variation in the genetic make-up of humans. Such polymorphisms can result in different disease risks. An example is differences in blood concentrations of high-density lipoprotein cholesterols (HDL-C) between people from North-West Europe and East Turkey due to genetic polymorphisms [11,12]. Studying the explanatory variables of migrant health is thus complex. The different factors of nature and nurture act on their own and in combination (interaction), i.e. in the context of gene-environment interaction.

### Migration as health transition

Some migrant populations experience a lower mortality than the indigenous populations, despite their, on average, lower socio-economic status. This mortality advantage can be substantial. Singh & Hiatt showed that migrants in the USA tend to experience an up to 30% lower mortality from common cancers, cardiovascular disease (CVD) and diabetes, relative to the non-migrant population [13]. In Germany, studies of labour migrants from southern Europe showed a lower overall mortality compared to the indigenous population [14].

These seemingly contradictory findings can be explained in terms of migration as a health transition: Many migrants entering Europe or the US from economically less-developed countries move from a society in an earlier phase of the health transition to a society in a more advanced phase [5]. These migrant populations thus experience an unusually rapid health transition, which affects their health situation. Two components of this health transition are relevant:

- Therapeutic component: Mortality due to infectious disease as well as maternal and child mortality decreases quickly after migration, due to better health care in the country of immigration compared with the country of origin.

- Risk factor component: Risk of infectious disease decreases due to better hygiene and environmental conditions (e.g. safe drinking water supply, nutrition). At the same time, new risk factors for chronic diseases (cancer, cardiovascular disease, diabetes etc.) emerge, such as smoking, western nutritional habits and physical inactivity. Chronic diseases become the major cause of death, but only after a lag period.

Migrants benefit from improvements in health care, hygiene and nutritional conditions almost immediately after migration. They are thus experiencing a fast decline of some morbidity and mortality risks they brought along. Other risks increase, but mostly over a longer time period. The typical mortality pattern in western countries is characterized by chronic diseases with a long lag time (latency period) between relevant exposures and the clinical disease manifestation. Risk factors for cardiovascular disease (CVD), for example, act over a long time during the life course and show their effects mainly in middle and older age. Initially, migrant populations tend to have lower morbidity and mortality rates from such chronic diseases compared with the population of the host country, but this advantage decreases over time (usually decades) with the adoption of a western life-style.

Another consequence of the health transition is that migrant populations may experience an increased mortality for specific diseases. In the study of Singh & Hiatt [13], for example, the mortality for stomach and liver cancer was higher than in the US population. Contributing causes of these increased risks could be that infections with *Helicobacter pylori *and hepatitis virus, respectively, are common in childhood in economically less-developed countries. Also, the risk for haemorrhagic stroke is increased among migrants from these countries [15], again associated with deprived living conditions before migration. Empirical findings such as these demonstrate that migrants might face specific exposures during childhood - a critical period - which contribute to health differentials in later life in the host country.

Not all empirical findings are perfectly in line with the model of migration as a health transition. An example is the increased risk (measured by mortality or health care utilization) for CVD among migrants from South Asia in the UK [16,17]. This increase occurs rapidly after migration. A possible explanation is offered by the adipose tissue overflow hypothesis [18]. It postulates a genetically determined higher risk for obesity in settings with calorically unrestricted nutritional intake. Here, a health transition does occur, but its effects are enhanced by a gene-environment interaction.

In contrast to South Asian migrants, ethnic German re-settlers from Eastern Europe (*Aussiedler*) have a lower CVD mortality compared with the indigenous German population [19]. No marked increase over time is visible at present; however, the post-migration observation period is still somewhat short. Factors such as social deprivation or high fat intake seem not yet to have an influence on CVD mortality in this migrant population.

In summary, the health situation of migrants is influenced by factors operating both in the country of origin as well as in the host country, and acting at various phases in the lives of migrants. The nature and importance of these factors has been difficult to determine and to quantify empirically, as previous models of migrant health have lacked a crucial element, namely an explicit time axis [2,6]. For this reason, we propose a new conceptual framework based on life course epidemiology. Such a concept should not only include the main factors acting on the health of migrant populations, but also make their temporal sequence explicit.

## Discussion

### Periods of migration

Migrant populations move through different phases of the health transition during their life course. The differences in exposures in this time line can be categorized in three major periods: (i) the period before migration, mostly including *in utero *exposures and the critical phase of early childhood, (ii) the migration process itself and (iii) the period after migration.

In period (i) migrants may be exposed to factors which are not - or are to a lesser degree - faced by the majority population in the host country. The resulting disease risks are constituted during critical periods in early childhood before migration and become manifest in later ages. Examples are higher risks for stomach cancer due to infection with *Helicobacter pylori*, or liver cancer due to hepatitis B or C. Another example may be the higher incidence of childhood leukaemia observed among Turkish children in Germany [20]: There is evidence showing that infectious exposures due to unusual population mixing (populations usually separated coming in contact with each other) modify the risk of acute lymphoid leukaemia [21]. In addition, factors might have existed that lead to the decision to migrate, such as exposure to war, terrorism, natural disasters or political repression. These factors can be substantial stressors and affect the physical and mental health of migrants in later life.

The process of migration itself - period (ii) - is a sensitive phase: the migration process produces stress, which might in turn increase the risks for specific psychiatric diseases or for CVD. In addition, in the period after the immigration process - period (iii) - migrants often live in poorer socioeconomic conditions than the indigenous population of the host country, which may increase disease risks by a process of accumulation. However, it should not be ignored that migrants might either have specific health benefits and resources [7], for example a high level of reciprocity in their communities [22], or more favourable health behaviour such as healthier nutrition or lower levels of smoking (Reiss et. al. 2010, Reeske et al. 2009) and alcohol consumption, which interact with the other risk factors and can result in lower risks for some diseases.

There is evidence that the risk of several chronic diseases, including stroke, allergic disease and cancer [23-25], is influenced by early childhood exposures. Migrants often face different exposures in their life course compared with the autochthonous populations of the host countries due to the different situation in their home countries (nutrition, hygiene, prevalence of infectious diseases, etc.). The inclusive consideration of these different influences and their time scale in a life course perspective, currently an important theme in Public Health/Epidemiology [8-10,26], is still missing in the research on migrant health.

### A life course approach

**Figure 1:**
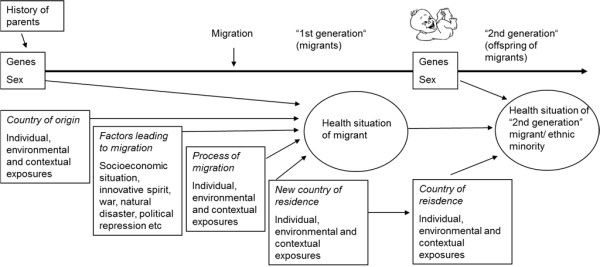
**Different exposures during the life course on the health of migrants**.

Figure 1 shows a conceptual framework for migration and health integrating the influence of exposures that migrants face during their life course. This approach shows the different exposures of first generation migrants along the three periods: in the country of origin, during migration and in the host country. Depending on the age at migration, exposures and critical periods such as early childhood, can fall into the period before, during, or after the migration, and accumulation of risks can take place in one period or over several periods. These different exposures, which act at different times during the life course, determine the disease risk of migrants. The model helps to understand why, for example, chronic diseases arise at different times and with different probability compared with the indigenous population of the host country. In particular, exposures in (early) childhood in the country of origin are included in the model. One example is the risk of obesity in adulthood, which is influenced by exposures in the prenatal phase: a restricted foetal growth and low birth weight, both common problems in many low-income countries from which migrants originate, increase the risk for obesity in adulthood [27]. The obesity risk of adult migrants is thus not only determined by their nutritional behaviour and physical activity in the host country. A high prevalence of obesity in migrant populations can also be the result of exposures during critical periods, e.g. *in utero *or early childhood. Another example is the possible role of infections during childhood on the risks for specific cancers. For example, the increased risks for lymphatic leukaemia among Turkish migrants in Hamburg, Germany, might be the result of a higher prevalence of exposure to Epstein-Barr virus in Turkey before migration [28]. Lower risks for gynaecological cancers among women of Turkish origin in the same study [28], in Sweden [29,30] or in North-Holland [31] might be explained by the lower prevalence of exposure to human papilloma virus in Turkey.

Other important factors such as family history, socioeconomic status, education and living conditions, as well as health behaviour like nutrition, physical activity, and alcohol and tobacco consumption can act during different periods of the migrants' life. Accumulations and interactions of exposures in migrants can occur in several ways, including the accumulation of "pack years", changed by migration due to availability of cigarettes [23,24], or complex gene-environment interaction and accumulation of high-calorie diet in the adipose tissue overflow hypothesis [16,18].

The health situation of the offspring of immigrants, the so called second generation, is also influenced by specific exposures. Differences in genetic endowment can be passed from parent to offspring, for example a darker skin type, and can result in specific health circumstances, such as lower risks for skin cancer or access barriers due to discrimination. Besides these genetic factors, parents may pass on other aspects to their offspring. For example, cultural beliefs, health behaviours (nutrition, smoking and alcohol consumption), reproductive factors and physical activity are influenced by the parents' lifestyle and behaviour. Specific cultural beliefs and behaviours of ethnic minorities may persist over generations. Socio-economic conditions of the parents determine the socio-economic situation of the offspring during childhood and can have a persisting influence in later life [32]. For these reasons, and due to their acculturation, the health situation of members of the second generation and of ethnic minorities differs from that of the autochthonous population. The health situation of the second generation is different to the health situation of first generation migrants, because the former did not face the exposures in the country of origin and during the migration process. Moreover, they may to some extent be more acculturated or segregated than their parents. The higher disease risks of migrants might converge with the risks of the autochthonous population in the second generation or in younger birth cohorts, as shown by studies about cancer risks among migrants in the Netherlands [33], British-Columbia, Canada [34], and Germany [28]. Alternatively, they may remain stable, as demonstrated in a study concerning the risk of skin cancer among Turkish migrants in Hamburg, Germany [28]. Acculturation could be a crucial factor for changes in health-related behaviour after migration or between generations. In the so called "dietary acculturation", for example, dietary habits of ethnic minorities are becoming less healthy due to increased intake of fat, sugar, salt and processed food [35]. In a "nutrition transition", dietary changes can be accompanied by changes in physical activity and obesity trends [36]. However, acculturation is difficult to measure [37] and the relationship between acculturation and changing health behaviours might differ according to the ethnic group examined and the measure of acculturation used [35,38].

The life course approach to migrant health research presented here takes into consideration the different factors acting over the life course of migrants that researchers have to consider when describing and interpreting the current health status of migrants. So far, not all aspects of this framework have been empirically confirmed at an appropriate level of evidence, or specifically for migrants (in some cases, convincing evidence is available from studies of non-migrants). For example, the influence of nutritional and hygienic conditions in early childhood on stroke and stomach cancer needs to be supported with further evidence from prospective migrant studies. Additional research on the associations and interactions of the environmental, genetic and behavioural factors and their changes during the life course would allow a more detailed understanding of the health situation of migrants and how it changes over time, both in absolute terms, and relative to the health of the majority population in the host country.

### Methodological issues

Ideally, an analysis of migrant health should include all aspects mentioned in Figure 1, i.e., genetic background, situation in the country of origin, situation in the host country, and the attributes of the individual. Only if all interacting factors are understood (and controlled for, if necessary), can the influence of a single factor be appropriately analyzed. We need a better grasp of these complex interactions to be able to analyze specific sets of factors in a more deterministic way.

### Difficulties of longitudinal designs

Further studies about migrant health should aim to analyze the influence and interaction of factors falling in the "nature" and "nurture" categories when comparing disease risks of migrants with those of indigenous populations. A focus should be on the timing and dynamics of exposures. This will make it possible not only to describe point prevalences or risks of diseases, but also to show periodic differences in disease development. Studies including all these factors require a longitudinal follow-up not only of a migrant population, but also of the population in the country of origin and in the host country. Such studies pose enormous methodological challenges [39]: An appropriate instrument to examine life course aspects is a prospective birth cohort; however, such studies are very difficult to implement with first generation migrants, because these persons would have to be included before migration while still in their countries of origin, and even before they even know that they might migrate in future. Given these obvious obstacles, a retrospective exposure assessment has been the way of choice in migrant studies so far. Clearly, studies attempting to retrospectively assess exposures and collect information about early childhood also face several problems, such as recall bias and missing data. These problems increase if data from an economically less-developed country of origin are needed. However, in some countries of origin, such as Turkey, the quality and quantity of health data is improving. New mortality and disease registries are being set up, thus providing new opportunities for transnational migrant health research [39]. In other countries of origin, there are still far too little data available for such studies.

### The challenge of selecting suitable comparison groups

Researchers need to consider against which population(s) the health status of a migrant population should be compared, in particular when transnational epidemiological studies are possible. Comparisons can be made relative to:

- the population of the host country

- the population of the country of origin

- migrant populations of the same origin which migrated to other host countries.

Each comparison answers a different research question relevant for life course epidemiology.

**Figure 2:**
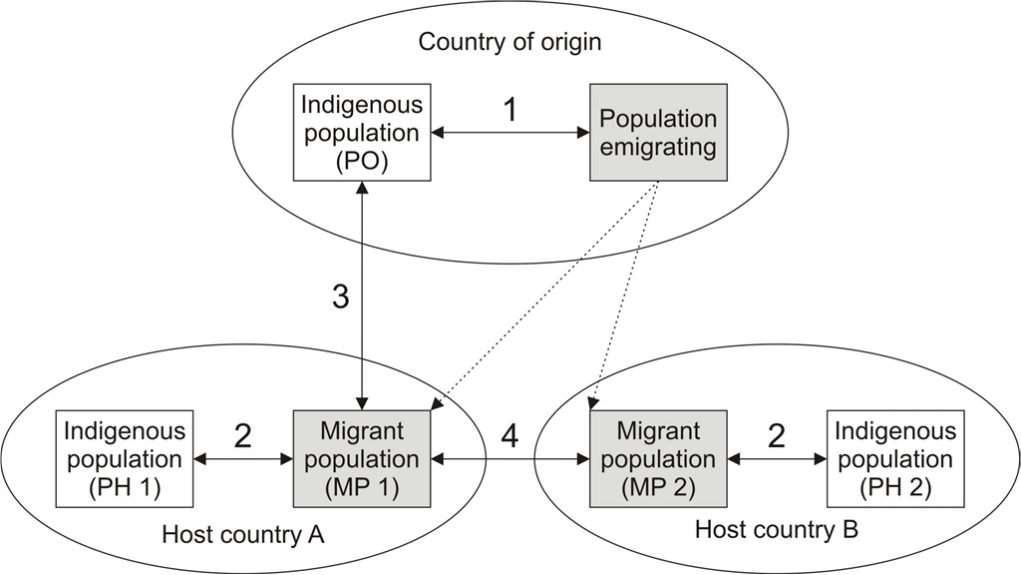
**Possibilities (1-4) to compare health of migrants with health of autochthonous (indigenous) populations**.

Figure 2 provides an overview of the possible comparisons of the health status of a migrant population. The choice of the appropriate comparison population depends on the study question: Comparison 1 enables studying the presence of a healthy migrant effect at the time of migration, that is, whether the population intending to emigrate is healthier than the general population of the country of origin (PO), a (self-) selection effect. Comparison 2 is the most common approach for analyzing the health of migrants, namely a comparison of the health of an immigrant population (MP1) to the health of the autochthonous population of the host country (PH1). It can help to investigate differences in exposures and in access to care. Comparisons 3 and 4 are approaches that are not used frequently at present. Comparison 3 investigates the health of a population who has already migrated (MP1) relative to the health of the population in the country of origin (PO), thus adding information about factors that are due to the migration process and the social and health situation in the host country. Comparison 4 adds information about the influence of factors specific to different host countries (MP1 vs. MP2). One example is differences in the structure of the respective health systems, which might affect access of migrants to care, and thereby their health status.

An ideal life course study of the health of migrants would comprise all four of these comparisons and so give new insights into the research questions raised above. Furthermore, including additional comparison groups besides the population of the host country will contribute towards producing more detailed information on the influence of exposures acting during the periods before, during and after the migration. While this ideal study is likely to remain elusive, it might be possible to implement some features in new collaborative studies. As is common for observational epidemiological studies, the complete set of factors and confounders relevant for health in a migrant's life course cannot be investigated in one comprehensive study. In any case, researchers need to keep in mind possible effects of the unmeasured factors during analysis, interpretation and dissemination of their results.

## Summary

The health of migrants is determined by factors that operate in different phases of their life course, and which may be considerably different from factors operating during the life course of members of the majority population of the host country. This strong temporal component is not sufficiently reflected by existing models of migrant health. Researchers studying migrant health should not only consider risks and exposures in the host country of migrants but also exposures during the migration process and in the country of origin. Studies of members of the second generation and of ethnic minorities should consider exposures of the parental generation and the possibility that specific behaviours and risks are passed on to the next generation. Studies taking this life course framework into account will provide new insights in the development of disease and the health situation of migrants. For example, analyses of the change of cancer risks over time since migration or between migrant generations can provide new insights into the causes of cancer, as well as critical periods, promoting factors, the influence of genes and environment, and latency periods of the different processes [40].

We believe that further discussion is essential for developing the theoretical framework of migrant health and for improving future empirical studies. Our own research focuses on extending the proposed concept of life course epidemiology in such a way that it can be applied to the offspring of migrants and ethnic minorities who have not themselves migrated.

## Competing interests

The authors declare that they have no competing interests.

## Authors' contributions

JS drafted the manuscript. JS and OR conceived the theoretical work. JS, HZ and OR contributed to the discussion and debate. All authors read, revised and approved the final version.
